# An observer tool to enhance learning of incoming anesthesia residents’ skills during simulation training of central venous catheter insertion: a randomized controlled trial

**DOI:** 10.1186/s12909-023-04915-9

**Published:** 2023-12-11

**Authors:** Dan Benhamou, Sarah Tant, Benoit Gille, Yannis Bornemann, Laura Ruscio, Karl Kamel, Chloé Dunyach, Bénédicte Jeannin, Maxime Bouilliant-linet, Antonia Blanié

**Affiliations:** 1 Centre de simulation LabForSIMS, Département de Recherche et Innovation Pédagogique en Santé, Faculté de médecine Université Paris Saclay, Le Kremlin-Bicêtre, 94275 France; 2 Département d’Anesthésie-Réanimation et Médecine Péri Opératoire, APHP, CHU Bicêtre, Le Kremlin Bicêtre, 94275 France; 3 CIAMS, Univ. Paris-Saclay, Université Paris-Saclay, Orsay Cedex, 91405 France; 4 CIAMS, Université d’Orléans, Orléans, 45067 France

**Keywords:** Simulation, Anesthesia, Technical skills, Observer tool, Observational learning, Central venous catheter, Education

## Abstract

**Background:**

Central venous catheter (CVC) insertion using simulation is an essential skill for anesthesiologists. Simulation training is an effective mean to master this skill. Given the large number of residents and the limited duration of training sessions, the active practice time is limited and residents remain observers of their colleagues for much of the session. To improve learning during observation periods, the use of an observer tool (OT) has been advocated but its educational effectiveness is not well defined.

**Methods:**

Incoming anesthesia residents were randomized to use an OT (i.e. procedural skill-based checklist) (OT+) or not (OT-) when observing other residents during a simulation bootcamp. The primary outcome was a composite score (total 60 points) evaluating CVC procedural skills rated immediately after the training. This score covers theoretical knowledge explored by multiple choice questions (MCQs) (/20), perceived improvement in knowledge and skills (/20), perceived impact on future professional life (/10) and satisfaction (/10). Measurements were repeated 1 month later. Residents in each group recorded the number of CVCs placed and their clinical outcomes (attempts, complications) during the first month of their clinical rotation using a logbook.

**Results:**

Immediately after training, the composite score was similar between the two groups: 45.3 ± 4.2 (OT+, *n* = 49) and 44.4 ± 4.8 (OT-, *n* = 42) (*p* = 0.323). Analysis of sub-items also showed no difference. Results at 1 month were not different between groups.

Analysis of the logbook showed no difference between groups. No serious complications were reported.

**Conclusions:**

The use of a procedural task-based OT by incoming anesthesia residents and used during CVC insertion simulation training was not associated with better learning outcomes, neither immediately after the session nor when re-evaluated 1 month later. The training at least once on simulator of all residents could limit the impact of OT. Further studies are necessary to define the place of OT in simulation training.

**Supplementary Information:**

The online version contains supplementary material available at 10.1186/s12909-023-04915-9.

## Background

Learning how to insert a central venous catheter (CVC) is essential for anesthesia residents and the use of simulation is recommended [1, 2]. Since 2017, this training has been integrated into the curriculum for incoming first year anesthesia residents in Île de France and combines a theoretical part (flipped classroom) and a practical part on a task simulator. Given the large number of residents and the limited duration of the training sessions, the time devoted to active practice on the simulator is reduced and residents often remain observers of their colleagues for a large part of the session.

The social learning theory proposed by Bandura and adapted to simulation states that vicarious learning occurs when observing others, one gets an idea of how behaviors are produced and how to reproduce them [3]. The effectiveness of observation in simulation based medical education is increasingly recognized [3, 4] but study results remain controversial [5–13]. A recent meta-analysis suggests that learning is more limited for an observer than for an active participant [4].

To increase the positive effects of simulation training when the learner is in the role of observer, some authors have proposed the use of an observer tool (OT) allowing observers to analyze the progress of the task performed by their colleagues [3, 14–16]. OTs are checklists describing key points to be achieved. However, data regarding the educational value of these OTs are limited. Studies have reported successful use of OT in the context of high-fidelity simulation for crisis management training in the operating room [15]. However, their educational benefit has not been tested during procedural simulation.

The objective of this procedural simulation study was to assess whether the use of an OT improves learning experience of CVC placement in a simulation environment.

## Methods

### Study description

This prospective and randomized study was conducted at the simulation center of Paris-Saclay University (LabForSIMS). Approval had been obtained from the Research Ethics Committee of the French Society of Anesthesia and Intensive Care Medicine (SFAR, CERAR: IRB 00010254–2021 – 196). The trial has been registered on ClinicalTrials.gov (Identifier: NCT05134818; 26/11/2021). The study was carried out with the use of the CONSORT tool adapted for simulation studies [17] and the GREET Tool for educational studies [18].

The training took place at the beginning of the first semester rotation during an initial training seminar (bootcamp) that is made mandatory for all incoming residents of the Paris area. The simulation session was preceded by a flipped classroom learning part (educational documents sent upstream including a video [19] while the practical CVC insertion session lasted 1 h30, with approximately 7 residents/1 instructor. The workshop consisted of three steps: (i) a short theoretical refresher, (ii) supervised practice using an echogenic rubber matrix and fluid-filled tubes simulating human soft tissue and vascular structures to learn needling with ultrasound, and (iii) supervised practice on a CVC simulator of the internal jugular vein (IJV) (chest trainer allowing ultrasound visualization (Ultrasound Catheter Insertion low fidelity Simulator, Kyoto Kagaku®, Ref: KKM93UB) (ultrasound machine, Mindray®, model TE7) (CVC, Arrow®, Ref: CV-04301). Each resident performed a complete CVC placement at least once on the simulator and was an observer when other residents were on the hot seat.

**Fig. 1:**
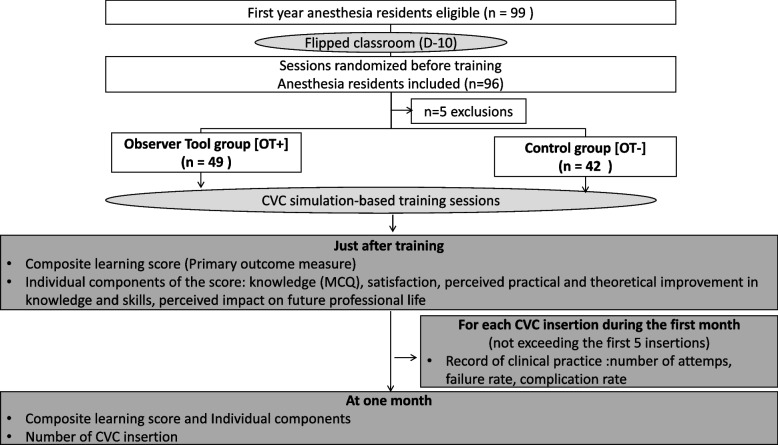
Study flow chart

After obtaining informed consent, residents were included (Fig. 1). Randomization was performed using the random function of the Excel© software) to obtain an equal number of sessions during which residents used (OT+) or not (OT -) the observation tool. Residents were informed that the tool was only used to reinforce learning, not for evaluation.

The OT has been adapted from a previously published checklist [20, 21] which describes in a logical and chronological manner the main steps for inserting a CVC. The initial checklist was validated by Hartmann et al. to assess performance during CVC insertion [21]. This checklist was adapted by removing 11 checklist items that were not achievable during our jugular CVC insertion training. The rationale for removing each item is detailed in Appendix 1. This modified checklist was already used by our team in a previous study [20].

### Assessment method

The primary outcome was a composite learning score [20] evaluating CVC placement skills immediately after training (levels 1–2-3 of the Kirkpatrick model [22]). The score (total 60 points) was composed of five sub-parts (objective and subjective assessments) evaluating: (i) theoretical knowledge based on multiple -choice questions (MCQs) (/ 20), (ii) perceived practical (/ 10) and theoretical (/ 10) improvement in knowledge and skills, (iii) perceived impact on future professional life (/ 10) and (iv) satisfaction (/ 10). All items were measured on a Likert scale from 0 to 10. Measurements were repeated 1 month later to assess retention.

Secondary outcomes included a separate analysis of each item of the composite score. During the first month of their clinical rotation, each resident recorded in a logbook the number of CVCs inserted (all access types), specifically those placed in the internal IJV. For the first insertion in the IJV, they had to report the number of attempts, insertion failure or complications (pneumothorax, arterial puncture, others) (levels 3 and 4 of the Kirkpatrick model [22]) (objective self-assessment).

A reminder was sent every 2 weeks to encourage recording of clinical data and a medical book was offered as incentive to each resident who had fully completed the final questionnaire.

### Statistical analysis

Assuming that an expected mean composite score would be 48 out of 60 in the control group, using a standard deviation of 6 points out of 60, and considering that an improvement of one standard deviation (difference accepted for studies in education [23]) a score of 54 out of 60 was expected in the intervention group. Using an alpha risk = 5% and a power of 90% with two-tailed tests), 22 residents had to be included in each group to observe a significant difference (https://biostatgv.sentiweb.fr/?module=etudes/sujets).

The composite score was compared immediately after training between the two groups (i.e., OT+ versus OT-). Secondary outcomes were analyzed by comparing the two groups immediately after training and 1 month after training.

Results are presented as mean ± standard deviation or percentage. Statistical analysis was carried out with JMP® software (Cary, NC 27513–2414, USA). Statistical comparisons used two-tailed Student’s t-test and analysis of variance for parametric and continuous variables, a Chi-square test for proportions, and a Wilcoxon test for non-parametric variables. A value of *p* < 0.05 was considered significant.

## Results

### Inclusion

In November 2021, 96 anesthesia residents participated in the study and were included (Fig. 1). Five of them were excluded because they had not answered the questionnaire. A total of 91 residents’ responses were analyzed: *n* = 49 in the OT+ group and *n* = 42 in the OT- group. Participant characteristics were not different between the groups and the perception of their theoretical knowledge before the training was similar (Table 1).

**Table 1:** Characteristics of participants

	OT+ (*n* = 49)	OT- (*n* = 42)	*P* value
**Male/female ratio (n)**	26/23	26/16	> 0.05
**Age (mean ± SD)**	24.3 ± 1	24.7 ± 1.8	0.219
**Perceived theoretical knowledge before training (mean /10 ± SD)**	4.4 ± 2.5	4.9 ± 2.2	0.296
**Perceived practical knowledge before training (mean /10 ± SD)**	3.2 ± 2.6	3.5 ± 2.5	0.587

Significant if *p* < 0.05. Results presented as mean ± standard deviation

Each resident was an active participant at least once and observed the performance of their peers six times (according to the mean number of residents in each group).

### Composite learning score after training

The composite learning score immediately after training was not significantly different between groups (*p* = 0.323) (Table 2). The analysis of the individual composite score items immediately after training did not reveal any significant difference between the two groups (Table 2).

**Table 2:** Composite learning score, comparison between the 2 groups and comparison between immediately after the training session and 1 month after the training session

	Immediately after training		At 1 month after training	
	OT+ *n* = 49	OT- *n* = 42	OT+ *n* = 24	OT- *n* = 29
**Composite learning score** (mean /60 **±** SD) ([A + B + C + D + E])	45.3 ± 4.2	44.4 ± 4.8	43.2 ± 4.9	42.9 ± 4.8
A- Perceived theoretical improvement in knowledge and skills *after* the training (mean /10 **±** SD)	6.9 ± 1.4	7.0 ± 1.4	6.0 ± 2.2	6.7 ± 1.8
B- Perceived practical improvement in skills *after* the training (mean /10 **±** SD)	8.0 ± 1.3	8.3 ± 1.0	8.0 ± 1.3	8.4 ± 1.2
C- Theoretical knowledge with multiple -choice questions (mean /20 **±** SD)	12.6 ± 2.7	12.0 ± 2.6	12.4 ± 2.5	11.9 ± 2.5
D- Satisfaction with the training session (mean /10 **±** SD)	9.1 ± 0.9	8.9 ± 1	8.7 ± 1.9	8.5 ± 1.4
E- Perceived impact on future professional life (mean /10 **±** SD)	8.6 ± 1.4	8.1 ± 1.4	8.0 ± 1.5	7.5 ± 1.8

All comparisons did not display any significant difference. Results presented as mean ± standard deviation

At 1 month, 53/91 responses (58%) were recorded (OT+: *n* = 24 and OT-: *n* = 29). The global score and the sub-items were similar between the 2 groups and comparable to those obtained immediately after training (Table 2).

### Internal jugular vein (IJV) catheter insertion during clinical practice

During the first month, residents in the two groups had inserted a similar number of IJV catheters: 2.9 ± 1.7 and 3.4 ± 2 respectively (OT+: *n* = 24 and OT-: *n* = 29) (Table 3). The analysis of the logbook did not show any difference in clinical outcomes (number of attempts, failures) and no serious complications were reported (Table 4).

**Table 3:** Number of CVC placed during the first month after training in the two groups

CVC insertions	OT+ (*n* = 24)	OT- (*n* = 29)	P value
**Number of CVC inserted **(all types of access)	3.4 ± 1.9	4 ± 2.4	0.30
**Number of CVC inserted **(internal jugular vein)	2.9 ± 1.7	3.4 ± 2	0.34

Significant if *p* < 0.05. Results presented as mean ± standard deviation

**Table 4:** Outcomes related to the first internal jugular catheters placed in clinical practice after the training session. Significant if *p* < 0.05

	OT+ (***n*** = 19)	OT- (***n*** = 21)	*p* value
**Number of attempts before success (n ± SD)**	1.4 ± 0.67	1.5 ± 0.75	0.48
**Help needed (%)**	13.7%	16.8%	0.73
**Failure rate (%)**	3.2%	6.3%	0.46
**Complications**	0	0	
**Perception of stress when placing a CVC (mean / 10)**	4.0 ± 2.2	5.0 ± 2.7	0.32

Significant if *p* < 0.05. Results presented as mean ± standard deviation

## Discussion

In this study, an observer tool (OT), based on chronological technical steps, was used with the aim of improving the training of incoming anesthesia residents to place a central venous catheter (internal jugular vein) during a simulation session. The use of an OT was not associated with better learning outcomes, immediately after the session and on reassessment, 1 month later.

As learning from observing is likely more limited than active participation during simulation sessions [4], some studies have tried to improve learning outcomes by using an OT [7, 14, 3, 24, 25]. OT is believed to allow more active learning by reinforcing attention during training [26]. This is based on [27] a theory suggesting that when two actions are performed simultaneously (in the present study observing the other resident and filling out the form), attention is increased, and even more so when the different elements to observe are frequent (in our study, the procedure was observed 6 times on average) [28]. In our study, and contrary to our expectations and previous reports [3, 25], no beneficial effect was demonstrated when using a technical skills-based OT. To our knowledge, this study is the only one that randomly studied the specific impact of an OT on the learning CVC insertion. In 2012, Kaplan et al. [7] provided observers with a “critical action checklist” including a set of technical and non-technical actions to improve patient care but all observers used the OT. The post-test evaluation, which was carried out using a questionnaire based on non-technical skills, showed no difference in the average score. The study by Stegmann et al. [14] studied the impact of an OT in 200 medical students trained with a mock patient with rectal bleeding and abdominal pain. The observers used or not a checklist targeting technical skills (performing a digital rectal examination) and non-technical skills (patient information, doctor-patient relationship). For each skill thirteen items were defined, and the observer had to evaluate the performance. A significant improvement in knowledge related to doctor-patient interaction was recorded among observers using an OT. Unfortunately, this study was not randomized. Another study [15] randomized anesthesia residents to use (or not) an OT while observing. The OT was based on cognitive aids for crisis management (i.e., emphasizing technical skills and medical knowledge). This study showed an increased acquisition of medical knowledge and skills when using an OT.

The absence of benefit reported in our present study could be explained by the fact that all the residents (with or without OT) had performed the task at least once on the task simulator. This active practice might have already improved their baseline skills (active education) thereby reducing the impact of the OT. Comparing groups of observers with or without OT but not practicing on the simulator might have potentiated the difference but ethical considerations precluded using this design. It is also possible that OT have differential impact based on clinical circumstances. They may be more appropriate for situations in which technical and non-technical skills are trained together [14–16]. In addition, as no guidance exists on how to build an effective OT, it is possible that the tool we constructed was suboptimal. It can also be suspected that asking residents to maintain their attention by repeatedly using the OT when other residents were performing the task created some cognitive fatigue, which might have counteracted the positive effect on learning. Finally, our main outcome was maybe not sensitive enough to detect subtle differences in procedural skill learning.

The available literature regarding the use of tools to increase observer learning is limited [3, 7, 14, 16, 24, 25] and more research is needed to define their pedagogical value. As shown above, study design is often of limited quality, making interpretation still uncertain.

Retention at 1 month was not only similar between groups but also similar to initial results. A similar number of procedures performed over the next month may explain this result. Comparing early and delayed results also revealed no difference. This was expected not only because trainees placed several CVCs during the following month, but also because the time interval was likely too short to identify any decay in knowledge. Any difference could also have been masked by the limited number of residents filling adequately their logbook, reducing the statistical power. We anticipated limited protocol compliance and therefore offered a well-known anesthesia book as incentive to complete the whole study. Offering a material or financial incentive is a well-known factor that can increase recruitment [29], complete participation in a program [30] or questionnaire response rate [31]. Unfortunately, this did not lead to full resident participation.

Transfer of learning (i.e. levels 3 and 4 of the Kirkpatrick model [22] was therefore assessed by analyzing the residents’ responses through their logbook ratings. No significant difference was found between the groups when considering the number of attempts before a successful puncture, the failure rate, the need for help and the level of stress. Interestingly, no serious complication was recorded. Although the perception of stress was moderate (5/10), the failure rate (23%) and the need for help (73%) were both high, confirming that a single simulation training session does not lead to expertise. Mechanical complications may arise from ultrasound-related pitfalls, even if the procedure is performed correctly [32]. Schmidt et al. suggested avoiding the pitfalls through hands-on training as well as appropriate curriculum and advice from clinically experienced physicians [33]. Hence, in our study, the initial part of the workshop was dedicated to basic learning (hand-eye coordination and needle visualization).

The strengths of this study included the fact that our study was prospective and randomized and that we used a previously validated CVC insertion grid [20, 21].

However, it also has several limitations. All participating residents (with or without OT) performed at least one CVC placement on the simulator during the session and this may have masked any additional effects of the tool.

The use of a composite score to assess learning might have limited the validity of our results. However, this score has been used in one of our previous studies [20] to assess acquisition of the CVC procedural skills through several levels (i.e. 1–2 and 3) of the Kirkpatrick model. This score mixed a subjective assessment (perceived learning) with an objective assessment (knowledge test with MCQs). In addition, it would have been difficult to use a study design in which external evaluation could have been used (organizational limitations of this mandatory training and the large number of residents). The first month questionnaire was partly a self-assessment but also contained some objective questions extracted from their real-life practice (i.e. number of CVCs inserted, number of attempts, failures and complications). As residents were working in 15 different hospitals, external evaluation of their clinical practice was not possible. Finally, this study did not include any pretest because of an expected risk of learning bias. Use of a randomized design and inclusion of residents with no previous experience of CVC insertion, however, confirmed the validity.

## Conclusion

An observer tool based on the technical steps needed to be performed when placing an internal jugular vein central catheter was used during training of incoming anesthesia residents during a simulation session but was not associated with better learning outcomes immediately after and 1 month later. Further studies are necessary to define the place of observer tools in medical education.

### Supplementary Information

**Additional file 1: Appendix 1.** Template describing the modified checklist for inserting a central venous catheter [21]

## Data Availability

The datasets generated during and/or analyzed during the current study are available in the figshare.com repository, Blanié, Antonia (2023): CVC observer tool study. Figshare. Dataset. 10.6084/m9.figshare.22323472.v1.

## Abbreviations

CVC: Central venous catheter
OT: Observer tool
IJV: Internal Jugular Vein
