# Supplementary material for: An observer tool to enhance learning of incoming anesthesia residents’ skills during simulation training of central venous catheter insertion: a randomized controlled trial
Source: BMC Med Educ. 2023 Dec 11;23:942. doi: 10.1186/s12909-023-04915-9 (PMC10714635; doi:10.1186/s12909-023-04915-9)
Supplement: Supplementary file 1 — Additional file 1: Appendix 1. Template describing the modified checklist for inserting a central venous catheter [21] [file 12909_2023_4915_MOESM1_ESM.docx]

## Appendix 1: Template describing the modified checklist for inserting a central venous catheter [21].

|  | **Procedural step** | **Done** | **Not done** |
| --- | --- | --- | --- |
| 1 | Can describe the indication of the procedure |  |  |
| 2 | Obtains patient consent |  |  |
| 3 | Selects the site according to the patient's characteristics and the clinical situation |  |  |
| 4 | Confirms the puncture site with the ultrasound |  |  |
| 5 | Selects the appropriate equipment, probe cover and protective gear. |  |  |
| 6 | Appropriate antiseptic application with CHX-alcohol |  |  |
| 7 | Proper patient positioning |  |  |
| 8 | Put a mask and a cap and then washes his (her) hands |  |  |
| 9 | Wears gown and gloves |  |  |
| 10 | Covers the patient with a large sterile drape |  |  |
| 11 | Covers the ultrasound probe with the sterile sheath |  |  |
| 12 | Anaesthetizes the skin of the whole puncture area |  |  |
| 13 | Prepares the infusion line(s) and plug all lines except the distal lumen |  |  |
| 14 | Re-identifies the vein with the ultrasound, check for compressibility and locate the carotid artery |  |  |
| 15 | Under ultrasound control, slowly advances the needle while maintaining aspiration |  |  |
| 16 | When the blood vessel is entered, confirms that it is the vein and not the artery |  |  |
| 17 | Holds the needle in place and insert the wire (remove the syringe or insert the leader into the Raulerson syringe). |  |  |
| 18 | Moves gently the wire forward and checks that it can be moved smoothly in both directions |  |  |
| 19 | Incises the skin with a scalpel |  |  |
| 20 | Removes the needle by maintain control of the wire |  |  |
| 21 | Inserts the dilator with a rotating movement and then removes it |  |  |
| 22 | Inserts the catheter over the guide and then extends the guide until it exits through the distal lumen |  |  |
| 23 | Advances the catheter to the correct distance (≤ 15 cm on the right side, ≤ 18 cm for the left side) |  |  |
| 24 | Removes the guide, checking that it is intact and occludes the distal lumen. |  |  |
| 25 | Confirms the correct intravenous situation by aspirating the blood and then flushing with a crystalloid solution |  |  |
| 26 | Attaches the catheter to the skin |  |  |
| 27 | Covers the catheter with a sterile dressing |  |  |
| 28 | Put materials and cutters in the appropriate bin |  |  |
| 29 | Confirms the correct position and absence of pneumothorax (usually chest X ray) |  |  |
| 30 | Informs the nurse that the line can be used |  |  |

Check done/not done depending on what is done or said by the operator (except grey lines)

CHX: chlorhexidine

Removal of 11 items from the checklist of Hartman et al [21]:

*Item 2*: Residents were asked to assume that the “patient” was consenting and that there was no need to explain again the indication and the flow of the procedure.

*Items 3, 4 and 7*: The CVC was placed on a phantom dummy for the right internal jugular vein. Residents had no choice regarding the site or the patient’s positioning. Similarly, it was understood that the vein and artery did not present any anatomical abnormality or contraindication to puncture, i.e. identification with ultrasound prior to installation for the central line was unnecessary.

*Item 5*: Residents were told to ask for the necessary material but did not choose its type. The type of equipment (catheter size and number of lines) depended on the availability in the simulation center.

*Item 13*: CVC lines were not connected to an infusion bag at the end of the procedure.

*Items 19, 26, 27*: Residents were asked to avoid doing any incision on the skin of the phantom in order to avoid damaging the material. Similarly, the fixation and dressing steps were discussed orally but not performed on the manikin.

*Items 30* Communication with the nurse in charge was not included
